# B-cell immune repertoire analysis of autoimmune neurological syndromes with anti-GAD65 antibodies

**DOI:** 10.3389/fneur.2025.1668617

**Published:** 2026-04-16

**Authors:** Tianya Dong, Siyuan Fan, Haitao Ren, Yinwei Sun, Hongzhi Guan, Jing Wang

**Affiliations:** 1 State Key Laboratory of Cognitive Science and Mental Health, Institute of Psychology, Chinese Academy of Sciences, Beijing, China; 2 Department of Psychology, University of Chinese Academy of Sciences, Beijing, China; 3 Department of Neurology, Peking Union Medical College Hospital, Chinese Academy of Medical Sciences and Peking Union Medical College, Beijing, China

**Keywords:** glutamic acid decarboxylase-65 antibodies, limbic encephalitis, cerebellar ataxia, stiff person syndrome, immune repertoire

## Abstract

**Background:**

Autoimmune neurological syndromes (AINS) associated with anti-GAD65 antibodies encompass a spectrum of disorders, including limbic encephalitis (LE), stiff-person syndrome (SPS), and cerebellar ataxia (CA). Despite the universal presence of anti-GAD65 antibodies across these syndromes, patients exhibit remarkable clinical phenotypic diversity. Exploring the overall B-cell receptor (BCR) profile of patients with anti-GAD65 AINS may provide clues to the molecular pathological mechanisms underlying this phenotypic diversity.

**Methods:**

We performed high-throughput B-cell receptor (BCR) heavy-chain sequencing (Illumina NovaSeq 6000) on peripheral blood samples from 9 anti-GAD65 AINS patients (7 LE, 1 SPS, 1 CA/SPS overlapping) and 11 healthy controls (HC). Analysis encompasses clonotype profiling, somatic hypermutation (SHM) rates, CDR3 physicochemical characterization, repertoire diversity, and machine learning-based VDJ feature identification.

**Results:**

Anti-GAD65 LE showed significantly reduced repertoire diversity versus HC (Chao1: *p*-adjusted = 0.0023; Hill: *p*-adjusted = 3.7 × 10^–^⁵), lower SHM rates (3.54% vs. 4.73%), and enrichment of hyperexpanded clones. Distinct IGHV/IGHJ usage was identified, notably decreased IGHV1-2 and IGHV1-46 with elevated IGHV3-7. A random forest classifier built on characteristic VDJ clonotypes achieved near-perfect diagnostic performance (AUC > 0.99). Cross-patient shared clonal clusters further highlighted disease-associated BCR convergence.

**Conclusions:**

BCR repertoire heterogeneity across anti-GAD65 AINS subtypes illuminates the immunological underpinnings of phenotypic diversity. The identified VDJ signatures and common clonotypes represent candidate biomarkers for precise diagnosis and rational targets for BCR-lineage-directed immunotherapy.

## 1 Introduction

Autoimmune neurological Syndromes (AINS) associated with anti-GAD65 antibodies demonstrate substantial phenotypic heterogeneity, including common neurological syndromes such as limbic encephalitis (LE), stiff-person syndrome (SPS), and cerebellar ataxia (CA) (1–4). Anti-GAD65 LE is a form of Autoimmune Encephalitis (AE) that occurs infrequently (3, 5, 6), and typically presents with subacute seizures, cognitive deficits, confusion, and behavioral changes (7, 8). SPS is the most prevalent GAD-associated neurological disorder, characterized by stiffness of the proximal muscles of the trunk and limbs due to sustained co-contractions of agonist and antagonist muscles (9–12). CA is the second most common GAD65-related neurological disorder (8). Truncal and limb ataxia were the most common clinical symptoms in these patients, accompanied by severe dysarthria and motor dysfunction that often overlap with SPS (4, 13). The overlapping and distinct clinical phenotypes among the patients may stem from similarities and differences in antibody epitopes or immune response patterns. Nevertheless, no conclusive direct evidence has emerged to validate this hypothesis.

The majority of autoimmune disorders involve a loss of immune tolerance toward self-antigens, leading to the production of autoantibodies (14). B-cell receptor (BCR) repertoire reflects the adaptive immune status of the individual, and its diversity is closely related to the pathogenesis of autoimmune diseases (15, 16). High-throughput sequencing-based BCR repertoire analysis has enabled the resolution of BCR features from multiple dimensions, such as clonotypes and somatic high-frequency mutations, and has become a powerful tool for studying this area (17, 18). In recent years, BCR sequencing (BCR-seq) technology has been used to explore potential diagnostic markers and pathogenic mechanisms for diseases such as anti-N-methyl-D-aspartate receptor encephalitis (NMDARE) and anti-leucine-rich glioma inactivation 1 protein encephalitis (LGI1E) (19–21). However, BCR studies for anti-GAD65 AINS are still extremely limited (3, 22–24). And the characteristics of the B-cell repertoires in patients with different clinical phenotypes (LE, CA, and SPS) and their association with disease heterogeneity remain to be elucidated.

Overall, it is essential to explore the disease-specific immune response patterns in anti-GAD65 AINS through a systematic investigation of the characteristics of BCR repertoire. In this study, we systematically analyzed the peripheral blood B-cell repertoires of patients with anti-GAD65 LE (*n* = 7), SPS (*n* = 1), CA with SPS (*n* = 1), and healthy controls (HC) (*n* = 11). Through clonotype analysis, amino acid profiling, diversity assessment, our research aims to elucidate the possible causes of the clinical heterogeneity of anti-GAD65 AINS. Meanwhile, identifying VDJ rearrangement and characteristic clones of anti-GAD65 AINS provides a theoretical foundation for developing phenotype-specific biomarkers, which could support more precise diagnostic and treatment strategies adapted to these specific conditions.

## 2 Materials and methods

### 2.1 Study design

The PBMC samples from 9 anti-GAD65 AINS patients were collected from November 2020 to December 2021, including 7 patients with anti-GAD65 LE, 1 patient with SPS, and 1 patient with CA overlapping SPS phenotype. PBMC samples from 11 HC were collected from December 2021 to February 2022. Approval for this study was granted by the Institutional Review Board of Peking Union Medical College Hospital (IRB JS-2422). All participants, or their legal representatives, provided written informed consent prior to enrollment. All patients with anti-GAD65 LE fulfilled the diagnostic criteria for autoimmune limbic encephalitis proposed by Graus et al. (6); SPS fulfilled the revised 2009 diagnostic criteria for SPS (25) and CA met the cerebellar ataxia clinical phenotype (26). Anti-GAD65 IgG antibodies in cerebrospinal fluid (CSF) or serum samples were tested for by indirect immunofluorescence (cell-based assay) with commercially available biochips (Euroimun, Lübeck, Germany). All cases tested positive for CSF anti-GAD65 antibodies and negative for other antibodies (the antibody spectrum of encephalitis is shown in Supplementary Table S1). Clinical information on the patients is summarized as shown in Table 1.

**Table 1:** Clinical features of studied subjects.

Category	Anti-GAD65 AINS (*n* = 9)	LE (*n* = 7)	CA (*n* = 1)	SPS (*n* = 1)	HC (*n* = 11)
Age (years)	35.2 ± 16.1	31 ± 13.0	63	37	27.8 ± 2.7
Female, *n* (%)	8 (88.9)	6 (85.7)	1 (100)	1 (100)	9 (81.8)
Tumor, *n* (%)	0 (0)	0 (0)	0 (0)	0 (0)	0 (0)
Decreased level of consciousness, *n* (%)	1 (11.1)	1 (14.3)	0 (0)	0 (0)	0 (0)
Psychiatric symptoms, *n* (%)	4 (44.4)	4 (57.1)	0 (0)	0 (0)	0 (0)
Seizure, *n* (%)	6 (66.7)	6 (85.7)	0 (0)	0 (0)	0 (0)
Memory deficit, *n* (%)	7 (77.8)	6 (85.7)	1 (100)	0 (0)	0 (0)
Limb stiffness, *n* (%)	1 (11.1)	0 (0)	0 (0)	1 (100)	0 (0)
Ataxia, *n* (%)	1 (11.1)	0 (0)	1 (100)	0 (0)	0 (0)
Antibody positivity in cerebrospinal fluid, *n* (%)	9 (100)	7 (100)	1 (100)	1 (100)	0 (0)
Antibody positivity in serum, *n* (%)	9 (100)	7 (100)	1 (100)	1 (100)	0 (0)
Abnormality on MRI, *n* (%)	5 (55.6)	5 (71.4)	0 (0)	0 (0)	0 (0)
Abnormality on EEG, *n* (%)	6 (66.7)	4 (57.1)	1 (100)	1 (100)	0 (0)
Sample-ID	GAD-04, GAD-07, GAD-11, GAD-12, GAD-13, GAD-14, GAD-15, GAD-16, GAD-17	GAD-04, GAD-12, GAD-07, GAD-13, GAD-15, GAD-16, GAD-17	GAD-11	GAD-14	HC-01, HC-02, HC-03, HC-04, HC-05, HC-06, HC-07, HC-10, HC-11, HC-12, HC-13

Anti-GAD65 AINS, anti-GAD65 autoimmune neurological syndromes; LE, anti-GAD65 limbic encephalitis; CA, anti-GAD65 cerebellar ataxia; SPS, anti-GAD65 stiff person syndrome; HC, healthy controls.

### 2.2 Sample processing and RNA extraction

Lymphocytes were isolated, collected, and counted from PBMC samples through density gradient centrifugation. The isolated lymphocytes were then either directly subjected to subsequent experiments or frozen in liquid nitrogen for future use. Total RNA was extracted from the lymphocytes of 9 patients with anti-GAD65 AINS using the Vazyme FastPure Cell/Tissue Total RNA Isolation Kit V2. Additionally, total RNA from 11 HC was extracted from isolated lymphocytes utilizing the Trizol extraction method.

### 2.3 High-throughput bulk sequencing of BCR heavy chains

Bulk sequencing of the BCR heavy chain was performed as follows: mRNA was reverse-transcribed using Oligo (dT) primers to generate complementary cDNA. Subsequently, target gene multiplex PCR was performed using primers specific to IGHV FR1 in the variable region and primers targeting the constant region of the BCR, including IGHM, IGHD, IGHA, IGHG, and IGHE. Sequencing libraries were constructed by incorporating universal primer PCR adapters compatible with the Illumina sequencing platform. Library quality was initially assessed using Qubit 4.0 and gel electrophoresis. Following this, libraries were analyzed using the Agilent 2,100 Bioanalyzer to evaluate DNA fragment integrity and determine insert sizes. Quantitative PCR (qPCR) was used to accurately quantify the effective concentration of each library. Libraries were then pooled into flow cells based on their concentrations and the required sequencing depth. Library clustering was performed using cBOT bridge PCR. Finally, paired-end 150 bp (PE150) sequencing was conducted on the Illumina high-throughput sequencing platform (NovaSeq 6,000) to generate the raw data.

### 2.4 Analysis of raw data

Raw data were analyzed using MiXCR (version 1.2.0) (27) to identify productive clones. The R package *immunarch* (version 4.2.0) was used to analyze gene usage preferences for IGHV and IGHJ. The somatic hypermutation rate of the BCR (FR3 region) was calculated using the Change-O (28) toolkit. Amino acid lengths of complementary-determining region 3 (CDR3) were analyzed and visualized using *immunarch*. The mean value was used to characterize the distribution of CDR3 amino acid lengths and the frequency of somatic hypermutation for intergroup comparisons, while the population average of normalized individual gene usage frequencies was employed to characterize the analysis of IGHV/IGHJ gene usage, so as to ensure the reliability of the statistical results.

Clonotype analyses showed the proportion of hyperinflated saprotrophs and rare clones, and *immunarch* was used to analyze the clonotypes of anti-GAD65 AINS. BCR diversity plays a crucial role in shaping the autoimmune response. This diversity is typically generated through rearrangement of the variable (V), diversity (D), and joining (J) genes at the complementary determining region 3 (CDR3) recombination junction (29)_._ Chao1 is a nonparametric asymptotic estimator of species richness (number of species in a population) (30). Hill numbers incorporate both species richness and relative abundance into a unified class of diversity indices, commonly referred to as Hill numbers of species (31). Many researchers have suggested the Hill Index as an index of “true diversity.” To compare diversity between multiple samples, we used *immunarch* to calculate Chao1 values and Hill numbers to assess the results of immunome diversity.

Meanwhile, the similarity of BCR (Jaccard-index) was analyzed and plotted using the R package *immunarch*. Sequences were clustered utilizing the Change-O pipeline to establish clone grouping thresholds for each donor. Our selected thresholds facilitated the calculation of the distance to the nearest neighbor, employing the SHazaM R package. The amino acid physicochemical properties of BCR were analyzed using *Alakazam* (32) (R package) in the Change-O pipeline.

### 2.5 Statistical analysis

Statistical analysis was performed using R software (version 4.2.0). Normally distributed data were expressed as mean ± standard deviation. The Wilcoxon test was employed for intergroup comparisons between the HC and the anti-GAD65 LE. As both the SPS and CA comprised only one sample each, descriptive numerical comparisons were employed instead. Data not following a normal distribution were summarized using medians (interquartile intervals). The VDJ clonotypes were screened using recursive feature elimination with cross-validation (scikit-learn-1.3), SVM analysis (the sklearn package in Python software) and k-fold cross-validation to assess the area under the receiver operating characteristic (ROC) curve.

## 3 Results

### 3.1 Basic analysis of the B-cell repertoire in PBMC from the anti-GAD65 AINS

**Figure 1:**
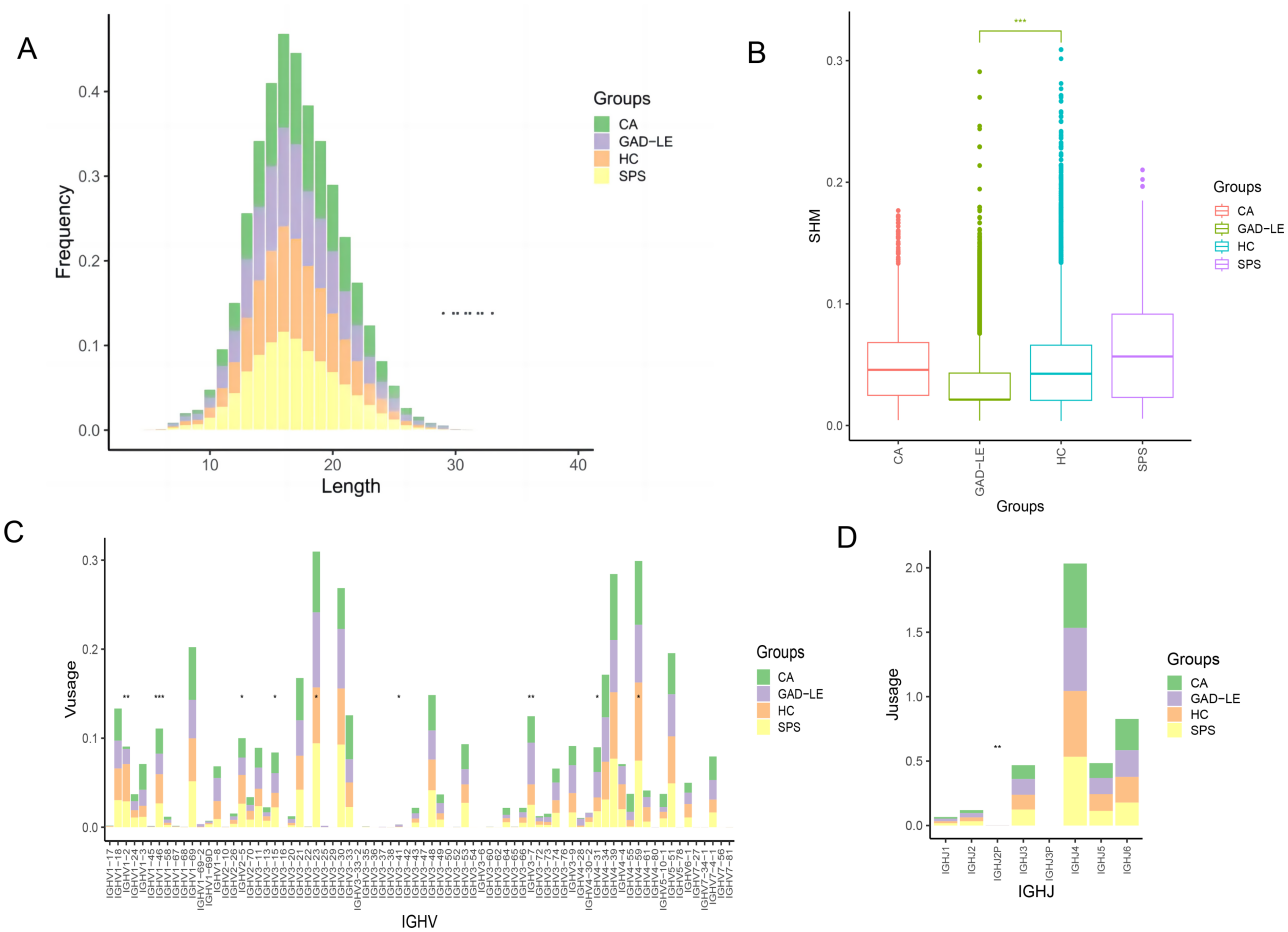
Basic analysis of the B-cell immune system in peripheral blood samples from anti-GAD65 AINS and HC groups. **(A)** CDR3 amino acid length distribution analysis. **(B)** Analysis of somatic hypermutation rates. **(C)** Analysis of IGHV gene usage preference. **(D)** Analysis of IGHJ gene usage preference. GAD_LE, anti-GAD65 limbic encephalitis; CA, anti-GAD65 cerebellar ataxia; SPS, anti-GAD65 stiff person syndrome; HC, healthy controls; ^*^p-adjusted <0.05, ^**^p-adjusted <0.001, ^***^p-adjusted <0.0001.

The distribution patterns of CDR3 amino acid lengths in both the anti-GAD65 AINS and HC groups were comparable and closely followed a Gaussian distribution (Figure 1A). The mean lengths of CDR3 amino acids in the anti-GAD65 LE, CA, SPS, and HC groups were 17.21, 17.50, 16.99, and 16.92 residues, respectively. There was a notable difference in the rate of somatic hypermutation between the anti-GAD65 AINS and HC groups (*p* < 2.22E-16). The mean somatic hypermutation rates of the anti-GAD65 LE, CA, SPS, and HC groups were 3.54, 4.72, 6.29, and 4.73%, respectively (Figure 1B). The mean values of the anti-GAD65 LE group were lower than those observed in the CA and SPS. Additionally, the level of IGHJ4 was found to be higher in the SPS than in the anti-GAD65 LE group, while the level of IGHJ6 was greater in the CA compared to that in the anti-GAD65 LE group (Figure 1C). After comparing the IGHV gene usage preference in the anti-GAD65 LE group to the HC group, the levels of IGHV1-2 (*p*_adj_ = 0.0008) and IGHV1-46 (*p*_adj_ = 0.00023) in the V genes were both significantly lower. In contrast, the levels of IGHV3-7 (*p*_adj_ = 0.0031) were markedly higher (Figure 1D).

### 3.2 Comparison of clonal amplification of diversity and clonality of B cell immune repertoire of anti-GAD65 AINS

**Figure 2:**
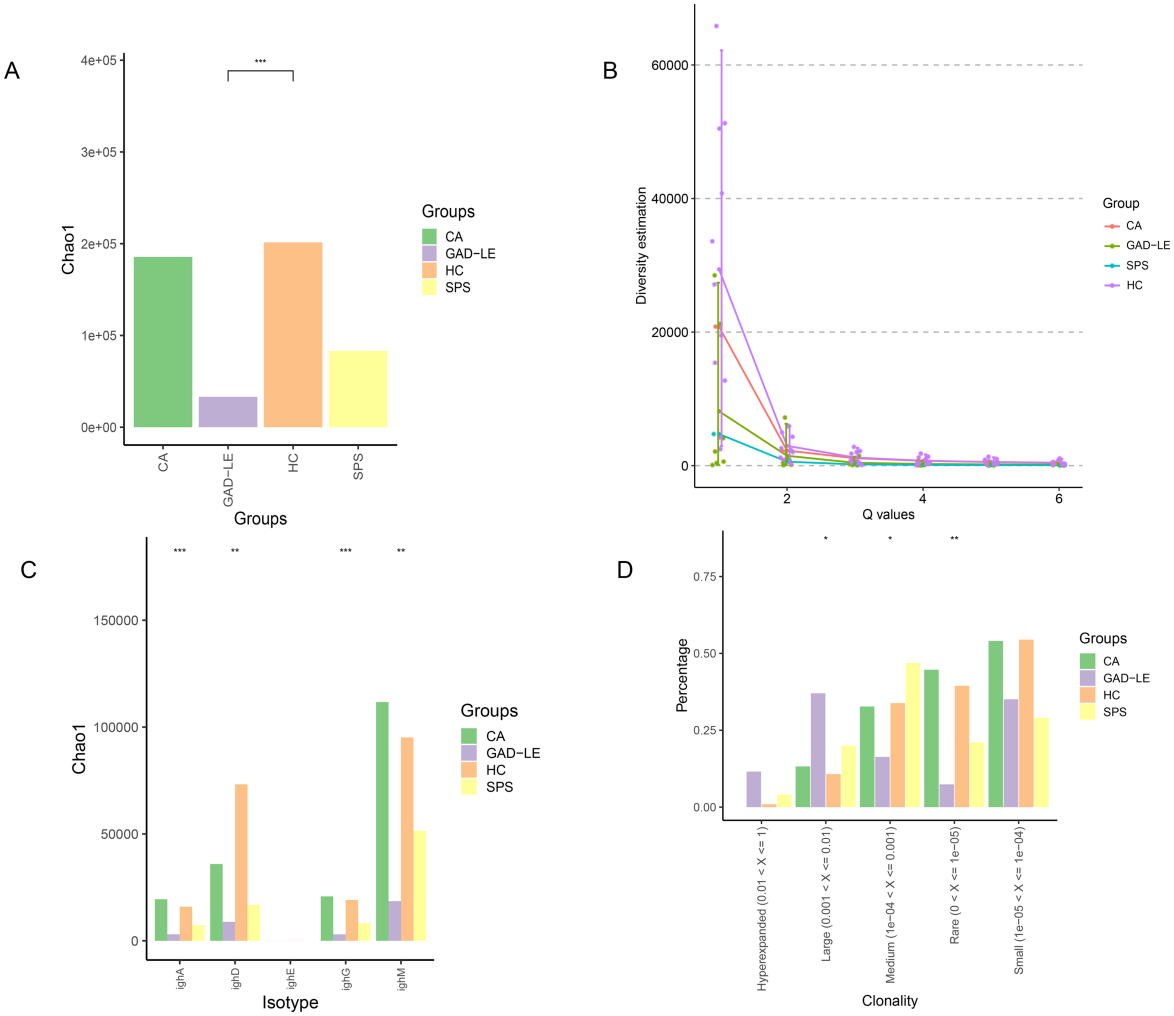
Comparison of clonal amplification, diversity, and clonality per isoform of the B-cell immune system in peripheral blood samples from anti-GAD65 AINS and HC groups. **(A)** Chao1 values for anti-GAD65 AINS and HC groups. **(B)** Hill values for the anti-GAD65 AINS and HC groups. **(C)** Chao1 values of the anti-GAD65 AINS and HC groups for different isotypes (IgA/IgD/IgE/IgG/IgM). **(D)** Clonality analysis for each group. GAD_LE, anti-GAD65 limbic encephalitis; CA, cerebellar ataxia; SPS, stiff person syndrome; HC, healthy controls; ^*^p-adjusted <0.05, ^**^p-adjusted <0.001, ^***^p-adjusted <0.001.

Normalized Chao1 analysis indicated that the HC group exhibited the highest repertoire diversity, which was markedly surpassed that of the anti-GAD65 LE group (*p*_adj_ = 0.0023) (Figure 2A). Meanwhile, the anti-GAD65 LE group had the lowest Chao1, while the CA group had Chao1 values slightly lower than those of the HC group. The results of the Hill coefficients also showed that the HC group had higher diversity than the anti-GAD65 LE group (*p*_adj_ = 0.000037) (Figure 2B). At the same time, the anti-GAD65 LE group had much lower diversity than the CA group. A comparison of the Chao1 per isotype revealed identical patterns of clonal expansion across each isotype among the anti-GAD65 LE, SPS, CA, and HC groups (Figure 2C).

The clonality of the BCR repertoire is shown in Figure 2D, with higher mean values of “Hyperexpanded Clones” (0.01 < frequency < 1) in the anti-GAD65 LE group, where significant clonal amplification occurred. There were more “Large Clones” (0.001 < frequency < 0.01) in the anti-GAD65 LE group than in the HC group (*p*_adj_ = 0.025). Conversely, “Rare Clones” (frequency ≤ 0.00001) were significantly less prevalent in the anti-GAD65 LE group than in the HC group (*p*_adj_ = 0.0019).

### 3.3 Analysis of immune repertoire amino acid physicochemical property

**Figure 3:**
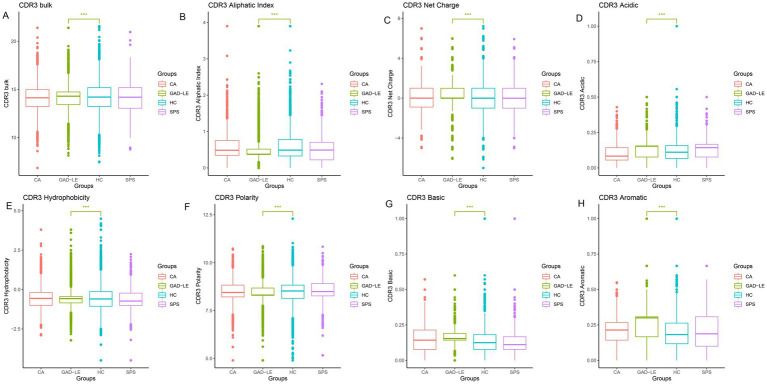
Comparison of amino acid physicochemical properties between the anti-GAD65 AINS group and the control group. **(A)** CDR3 bulk, average bulkiness. **(B)** Aliphatic index, normalized aliphatic index. **(C)** Charge, normalized net charge. **(D)** Aliphatic group, normalized aliphatic index. **(E)** Hydrophobicity, grand average of hydrophobicity. **(F)** Polarity, average polarity. **(G)** Basic, basic side chain residue. **(H)** Aromatic, aromatic side chain content. GAD_LE, anti-GAD65 limbic encephalitis; CA, cerebellar ataxia; SPS, stiff person syndrome; HC, healthy control; ^*^p-adjusted <0.05, ^**^p-adjusted <0.001, ^***^p-adjusted <0.001.

In the study of insulin resistance in mice, it was found that the IR of pathogenic mice not only changed in diversity, but also in the characteristics of charged and polarized amino acids in the CDR3 region. The amino acid properties of CDR3 are frequently used to characterize CDR3 binding to corresponding antigens, so we analyzed the differences in amino acid characteristics of CDR3 sequences from the anti-GAD65 AINS and HC groups (shown in Figure 3). These characteristics include gravy, grand average of hydrophobicity; bulkiness, average bulkiness; polarity, average polarity; aliphatic, normalized aliphatic index; charge, normalized net charge; acidic, acidic side chain residue; basic, basic side chain residue; aromatic, aromatic. In addition to the amino acid identity of polarity, the amino acid identities of the CDR3 sequences of the anti-GAD65 LE and HC groups were significantly different (*p* < 0.05).

### 3.4 Characteristics of VDJ rearrangement in anti-GAD65 AINS

**Figure 4:**
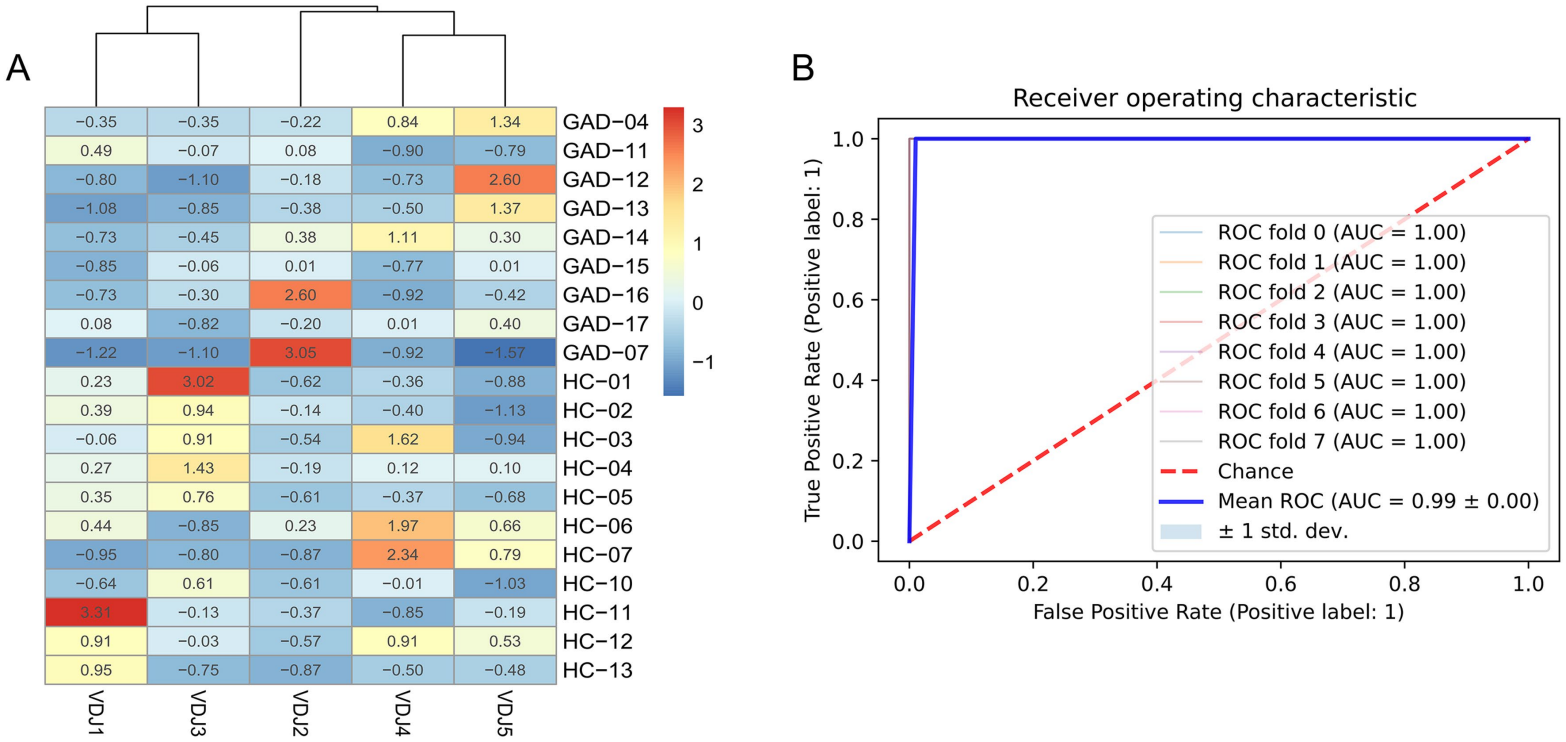
Differentially expressed VDJ feature combinations and anti-GAD65 AINS communality analysis. **(A)** Heat map of the expression of the screened feature VDJ combinations. **(B)** Random forest model classification of anti-GAD65 AINS and HC groups, with subject operating characteristic (ROC) curves assessing their performance. The colored area shows the 95% Confidence Interval (CI) of the curves. GAD_LE, Anti-GAD65 limbic encephalitis; CA, cerebellar ataxia; SPS, stiff person syndrome; HC, healthy controls.

We then screened for characteristic VDJ clonotypes of the anti-GAD65 AINS and HC groups using the circular feature method. A series of VDJs were identified, including IGHV2-5 IGHD6-13 IGHJ4, IGHV3-23 IGHD3-22 IGHJ4, IGHV4-59 IGHD3-3 IGHJ4, IGHV1-2 IGHD3-10 IGHJ4, IGHV3-30 IGHD3-10 IGHJ4 (Supplementary Table S2). We normalized the frequency results (Supplementary Table S2) and plotted a heat map (Figure 4A).

We further generated a heat map of VDJ recombination frequency curves and observed a significant difference between the anti-GAD65 AINS and the HC groups (PERMANOVA, *p*_adj_ < 0.05). Additionally, we trained a random forest model to evaluate the efficacy of BCR curves in differentiating between anti-GAD65 AINS and normal subjects. In the ROC curve analysis, a set of feature clones demonstrated high performance in identifying anti-GAD65 AINS (AUC > 0.99, Figure 4B). These results suggest the potential for developing BCR biomarkers for the early diagnosis of anti-GAD65 AINS.

### 3.5 Clustering analysis of anti-GAD65 AINS

We performed clustering analysis by Chang-O, a software that assumes antibodies with the same V and J genes in the heavy chain and a given CDR3 amino acid sequence identity tend to target the same antigens and epitopes than other BCRs. HC samples yielded 35,626 clusters from 138,894 sequences, which included 14,324 pairs of clustered clones co-existing across multiple HC individuals (*n* > 1). The results are presented in Supplementary Table S3.

Anti-GAD65 AINS samples yielded 7,786 clusters consisting of 170,176 sequences. The results are presented in Supplementary Table S4. We screened 48 pairs of clusters co-existing across multiple anti-GAD65 AINS individuals (n ≥ 2) (Supplementary Table S5) and found that 2 pairs of clonal clusters co-existed in 3 anti-GAD65 AINS individuals. The prevalence of these BCR clusters indicates their commonality among individuals. Generally, the frequency of patient-specific sequences is lower than that of the shared clones found in healthy populations.

## 4 Discussion

The innate and adaptive immune systems needs to function efficiently and interact with B cells to produce a diverse and functional BCR repertoire in response to antigenic stimulation (33). To our knowledge, our research is the first to systematically compare at the BCR level the BCR repertoire characteristics of three anti-GAD65 AINS with HC, revealing the unique immunological patterns of these diseases.

Patients with anti-GAD65 LE demonstrated a significantly lower rate of somatic mutations in the B-cell repertoire compared to the HC, CA, and SPS groups. Similar findings were found in previous studies of Autoimmune Encephalitis, where NR1-positive sequences from NMDARE patients were more likely to have a low or no mutation rate (34, 35). Meanwhile, the BCR repertoire from LGI1E was observed to exhibit a lower mutation rate compared to that of the HC group (21). The low mutation rate may reflect the production of antibodies by short-lived plasma cells outside the germinal center or be related to the breakdown of immune tolerance due to epitope spreading.

Compared to the HC group, the anti-GAD65 AINS groups showed a reduced diversity of B-cell repertoire. The anti-GAD65 AINS groups possessed fewer rare clones and more large clones compared with the HC group, suggesting that anti-GAD65 AINS status can dramatically affect the immune system by impairing BCR diversity in patients. The immune repertoire diversity of the LGI1E and NMDARE also showed a similar decrease (15). Furthermore, similar conclusions were drawn in immune repertoire studies of systemic lupus erythematosus (SLE), which employed next-generation sequencing to evaluate the T-cell repertoire in peripheral blood of SLE patients. The results showed that TCR diversity was significantly lower among SLE patients than in the HC group (36, 37). This indicates that a reduction in immune repertoire diversity may be a common characteristic of autoimmune responses.

Significant differences in CDR3 amino acid characteristics between the anti-GAD65 AINS and HC groups provide a theoretical basis for further searches of characteristic anti-GAD65 AINS sequences. Despite the limited sample size, the characteristic VDJ combinations (e.g., IGHV3-7/IGHJ4) identified through a machine learning model in this study demonstrated remarkable ability to distinguish anti-GAD65 AINS patients from HC (AUC > 0.99). This finding suggests that these clonotypes possess significant potential as diagnostic markers. Furthermore, shared clonal clusters (e.g., IGHV2-5/IGHD6-13/IGHJ4) identified in anti-GAD65 AINS patients via clustering analysis may represent BCR sequences specific to the GAD65 antigen. Functional validation of these clusters could facilitate the development of targeted therapeutic strategies.

We observed that the immune repertoire against GAD65_LE may exhibit similarities to that against LGI1E (21): characterized by reduced hypermutation frequency, diminished diversity, and increased clonal overlap. This finding may explain the observation that three cases of anti-GAD65 LE exhibited metabolic alterations in the basal ganglia. Such basal ganglia metabolic changes are recognized as characteristic features of LGI1E (3). However, significant differences persist between anti-GAD65 LE and anti-LGI1E antibodies. This apparent contradiction may stem from their distinct subcellular localization of target antigens: anti-GAD65 antibodies bind intracellular antigens, whereas anti-LGI1 antibodies target cell surface proteins. This suggests that differential antigen localization may influence clinical phenotypes by modulating same B-cell activation pathways (29).

In summary, our comparison of three anti-GAD65 AINS immune repertoire subtypes revealed reduced diversity across all subtypes. However, variations in hypermutation frequency and clonotype patterns were observed between them. Specifically in anti-GAD65 LE, we identified lower hypermutation frequency, a significantly increased number of hyperexpanded clones, and distinct physicochemical properties of amino acids compared to SPS and CA. Furthermore, leveraging the immune repertoire features of anti-GAD65 AINS, we identified characteristic VDJ combinations and public clonotype clusters. These findings provide critical clues for constructing disease-specific clones in anti-GAD65 LE and discovering biomarkers to facilitate timely diagnosis and treatment of these disorders.

Moreover, our identification of signature VDJ combinations and common clonal clusters provides a foundation for precision medicine strategies targeting pathogenic B-cell clones. Their high diagnostic specificity (AUC > 0.99) highlights their role in disease, supporting a shift from broad immunosuppression to targeted clonal elimination (e.g., via CAR T-cells or bispecific antibodies). This would eradicate pathogenic antibody sources while sparing healthy immunity. Additionally, leveraging these clonal sequences could aid in developing tolerance vaccines. Collectively, this advances the therapeutic paradigm for anti-GAD65 autoimmune neurological disorders toward BCR-lineage-based targeted therapies, offering a promising path to profound and sustained clinical remission.

However, the small sample sizes for SPS and CA in this study may have limited our statistical power to detect subtle differences between these subtypes and could potentially affect the generalizability of the observed BCR lineage characteristics. Future research directions should encompass the following: (1) expanding the cohort to validate the generalizability of phenotype-specific differences; (2) elucidating the interaction between BCR characteristics and T-cell helper signaling through integrated single-cell sequencing; (3) investigating the dynamic differences between CSF and peripheral blood BCR repertoires to clarify the mechanisms underlying antibody penetration across the blood–brain barrier; and (4) developing highly sensitive diagnostic reagents based on characteristic clonotypes identified in this study. These initiatives will offer new insights into the pathogenesis of anti-GAD65 AINS and provide a theoretical foundation for personalized immunotherapy strategies, such as targeting B-cell depletion or clonal clearance.

## Data Availability

The original contributions presented in the study are included in the article/Supplementary material, further inquiries can be directed to the corresponding authors.

## Supplementary material

SUPPLEMENTARY TABLE S1 The antibody spectrum of encephalitis.

SUPPLEMENTARY TABLE S2 VDJ clonotypes with significant difference among the anti-GAD65 AINS and HC groups.

SUPPLEMENTARY TABLE S3 BCR clusters in HC groups.

SUPPLEMENTARY TABLE S4 BCR cluster in GAD-AINS groups.

SUPPLEMENTARY TABLE S5 BCR common clonal clusters cluster in HC groups.
